# Gestational age-specific normative values and determinants of serum progesterone through the first trimester of pregnancy

**DOI:** 10.1038/s41598-021-83805-w

**Published:** 2021-02-18

**Authors:** Chee Wai Ku, Xiaoxuan Zhang, Valencia Ru-Yan Zhang, John Carson Allen, Nguan Soon Tan, Truls Østbye, Thiam Chye Tan

**Affiliations:** 1 Department of Obstetrics and Gynecology, KK Women’s and Children’s Hospital, 100 Bukit Timah Road, Singapore, 229899 Singapore; 2 Duke-National University of Singapore Medical School, 8 College Road, Singapore, 169857 Singapore; 3 Yong Loo Lin School of Medicine, National University of Singapore, NUHS Tower Block Level 11, 1E Kent Ridge Road, Singapore, 119228 Singapore; 4 Centre for Quantitative Medicine, Duke-National University of Singapore Medical School, Academia, 20 College Road, Singapore, 169856 Singapore; 5 Lee Kong Chian School of Medicine, Nanyang Technological University, 11 Mandalay Road, Singapore, 308232 Singapore; 6 School of Biological Sciences, Nanyang Technological University, 60 Nanyang Drive, Singapore, 637551 Singapore; 7 Department of Community and Family Medicine, Duke University Medical Center, 2200 West Main Street, Durham, NC 27710 USA

**Keywords:** Predictive markers, Health care

## Abstract

Progesterone is a steroid hormone that is critical for implantation and maintenance of pregnancy, and low levels are associated with higher miscarriage risk. However, little is known about its trajectory during early pregnancy. We sought to determine the gestational age-specific normative values of serum progesterone on a week-by-week basis, and its associated maternal and fetal factors, during the first trimester of a viable low-risk pregnancy. A cross-sectional study was conducted at KK Women’s and Children’s Hospital from 2013 to 2018. 590 women with a single viable intrauterine low-risk pregnancy, between gestational weeks 5 and 12, were recruited. Serum progesterone showed an increasing trend during the first trimester, with a transient decline between gestational weeks 6–8, corresponding to the luteal–placental shift. Lowest levels were seen at week 7. Maternal age, BMI, parity, gestational age and outcome of pregnancy at 16 weeks’ gestation were found to be associated with progesterone levels. Normative values of serum progesterone for low-risk pregnancies would form the basis for future work on pathological levels of serum progesterone that may increase risk of miscarriage. Larger studies are required to validate these normative values, and personalize them to account for maternal age, BMI, parity and gestational age.

## Introduction

Progesterone is a steroid hormone critical for the establishment and maintenance of early pregnancy. It prepares the endometrium for blastocyst implantation, sustains decidualization, reduces uterine contractility and promotes maternal immune tolerance to the fetal semi-allograft^1^. Many studies have reported a significantly higher risk of adverse pregnancy outcomes in women with low serum progesterone during pregnancy^1–4^. Excess serum progesterone, on the other hand, was reported to significantly suppress expression levels of decidualization markers in a dose-dependent manner and compromise embryo decidualization^5^.

In order to meaningfully interpret measured values of serum progesterone during early pregnancy, it is important to first establish gestational age-specific normative serum progesterone values to form a basis for reference and comparison. However, despite its pivotal role during early pregnancy, reference range studies of serum progesterone are predominantly trimester-specific and have wide ranges that are not clinically useful^6,7^. Furthermore, although progesterone has been reported to exhibit an overall increasing trend during pregnancy^2,8^, little is known about its trajectory during early pregnancy. Most importantly, women with low levels of progesterone have a greater risk of miscarriage^1–4^, and these patients could form an at-risk group for miscarriage with greater need for appropriate counselling or medical intervention.

Progesterone is secreted primarily by the corpus luteum, with the placenta eventually taking over if pregnancy occurs. This transition in progesterone production from the corpus luteum to placenta is known as the luteal-placental shift, and it occurs between weeks 6 and 8 of gestation. This period has been determined by earlier studies where corpus luteum removal prior to week 7 of gestation resulted in an immediate fall in serum progesterone levels and eventual miscarriage, while corpus luteum removal after week 9 of gestation resulted in pregnancy survival^9,10^. A few studies have suggested a trajectory where progesterone level starts decreasing around gestational week 5, reaching a nadir between weeks 6 and 8 corresponding to the luteal-placental shift, before increasing thereafter^11,12^. However, these studies are limited by small sample sizes as well as generalizability, for example by including only anovulatory women who conceived after induction of ovulation with gonadotrophins.

The primary aim of this study was to establish gestational age-specific normative values and a trajectory of serum progesterone in the first trimester of a viable low-risk pregnancy. The secondary aim was to determine maternal and fetal factors associated with serum progesterone levels in this group of low-risk women. We also aim to compare the serum progesterone levels in women who had viable pregnancies with women who had a miscarriage by 16 weeks. Establishing normative values of progesterone in early pregnancy has far reaching implications. Patients whose serum progesterone lies beyond the normal range, especially those with extremely low levels, should be brought to the attention of clinicians, as they represent a group of women who are potentially at risk of miscarriage, hence requiring closer monitoring or medical intervention.

## Methods

### Study design

This cross-sectional study was conducted from 1 January 2013 to 31 January 2018 at KK Women’s and Children’s Hospital (KKH), the largest maternity hospital in Singapore. The study involved one single progesterone measurement of each patient at presentation to determine the trajectory of serum progesterone in the first trimester.

### Study participants and eligibility

Study participants were pregnant women aged 21 and above presenting for routine antenatal screening at KKH clinics. Inclusion criteria were a single intrauterine pregnancy between weeks 5 and 12 of gestation, confirmed and dated via ultrasonography, with no pregnancy-related per vagina bleeding. Dating was performed via measurement of the crown-rump length. Gestational week was defined as gestational week 5 (week 5.0–5.9), gestational week 6 (week 6.0–6.9) and so on. Women with multiple gestations, previous episodes of pregnancy-related per vagina bleeding or those treated with progesterone in the current pregnancy, women diagnosed with an inevitable miscarriage, missed miscarriage, blighted ovum or planned pregnancy termination were excluded. To restrict the analysis to viable pregnancies, study participants with spontaneous miscarriage by week 16 of gestation were also excluded from the main analyses. Pregnancy outcome was determined via a phone call to study participants at week 16 of gestation and clinically confirmed to verify pregnancy status.

### Serum measurements

Serum progesterone level was obtained from blood samples of eligible study participants taken at presentation. Individual blood sample was collected in plain tubes and centrifuged for 10 min at 3000*g* within 2 h of collection. Samples were stored at − 4 °C prior to being tested. Serum progesterone level was subsequently measured in the KKH clinical laboratory using a commercial ARCHITECT progesterone kit (Abbott, Ireland), according to manufacturer’s instructions^13^.

### Maternal and fetal characteristics

Information on pregnancy characteristics were obtained to determine maternal and/or fetal characteristics that influence normative values of serum progesterone in the first trimester of a viable low-risk pregnancy. Information on maternal characteristics were obtained from questionnaires administered by an investigator either in English or Chinese, while information on the fetal characteristics were obtained either from clinical notes or during subsequent follow-up visits. Prior to the start of patient recruitment, a literature review was conducted to identify potential pregnancy characteristics that may influence serum progesterone levels. These include gestational age, maternal characteristics such as Body Mass Index (BMI), age, parity, smoking status and ethnicity, as well as fetal characteristics such as gender. Maternal BMI at presentation was taken as a proxy for pre-pregnancy BMI, calculated as weight^14^/height (m)^2^ and stratified according to Asian-specific World Health Organization definitions of underweight (< 18.5 kg/m^2^), normal (18.5–< 23 kg/m^2^), overweight (23–< 27.5 kg/m^2^) and obese (≥ 27.5 kg/m^2^)^15^. Parity was defined as either nulliparous women who had never given birth, or parous women who had at least one full-term pregnancy previously.

### Statistical analysis

Quantile regression was performed to obtain the 2.5th, 10th, 50th, 90th and 97.5th percentiles of serum progesterone level versus week of gestation. Restricted cubic splines with knots at 6, 8 and 10 weeks were used to approximate the shape of the serum progesterone trajectory through gestation. Analysis of variance using least squares means was then used to test for significance of the serum progesterone distributions between individual gestational weeks.

Associations between maternal or fetal characteristics and log-transformed serum progesterone values were analysed using univariate analysis and a subsequent multiple linear regression analysis was performed to adjust for confounding factors. Serum progesterone levels in women who had miscarried by 16 weeks were compared with women with viable pregnancies at 16 weeks.

In all analyses, a p value of < 0.05 was considered statistically significant. SAS software version 9.4 (SAS Institute Inc., Cary, NC, USA) was used for statistical computations.

### Ethical approval

This study was reviewed and approved by the Singapore Centralized Institutional Review Board (CIRB 2016/3093). Written informed consent was obtained from all participants before participating in this study. All research described in this manuscript was performed in accordance with relevant guidelines and regulations.

## Results

**Figure 1:**
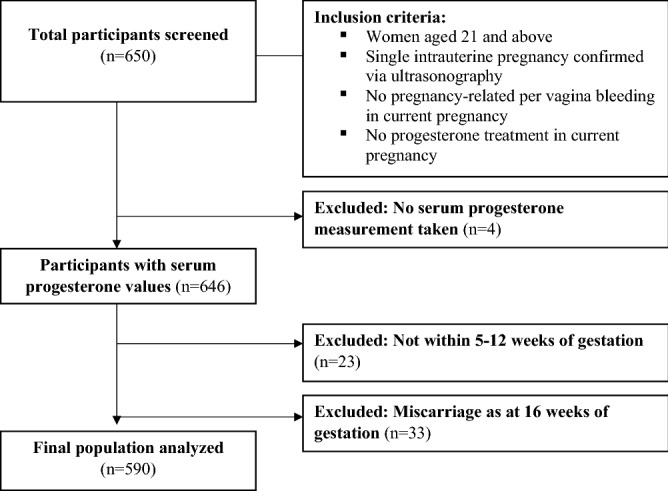
Consort diagram of study participants used to establish normative values of serum progesterone.

The final study population analysed comprised of 590 pregnant women (Fig. 1), after 60 women were excluded according to our exclusion criteria. The descriptive characteristics of the participants are shown in Table 1. Mean maternal age was 30.7 years, and the median gestational age at recruitment was 8.7 weeks. Majority of the participants were of Chinese ethnicity (48.8%), of normal BMI, and between 18.5 and < 23 kg/m^2^ (48.0%). Amongst the participants with information at delivery, 49.8% had a female baby and 50.2% had a male baby (missing data n = 134).

**Table 1:** Maternal and fetal characteristics of the study participants.

Characteristic	% (n) Total = 590
**Maternal characteristics**	
Age (years)	
< 25	4.4 (26)
25–< 30	36.1 (213)
30–< 35	41.7 (246)
35–< 40	15.9 (94)
≥ 40	1.9 (11)
Ethnicity	
Chinese	48.8 (288)
Malay	13.9 (82)
Indian	15.1 (89)
Others	22.2 (131)
BMI category*	
Underweight (< 18.5 kg/m^2^)	9.8 (58)
Normal (18.5–< 23 kg/m^2^)	48.0 (283)
Overweight (23–< 27.5 kg/m^2^)	25.9 (153)
Obese (≥ 27.5 kg/m^2^)	16.3 (96)
Number of children	
None	51.7 (305)
1 or more	48.3 (285)
**Fetal characteristics**	
Gender^#^	
Female	49.8 (227/456)
Male	50.2 (229/456)

*BMI* Body Mass Index.

*BMI taken at presentation, stratified according to Asian-specific World Health Organization definitions.

^#^Missing data for fetal gender at delivery, n = 134.

### Normative values and trajectory of serum progesterone

**Figure 2:**
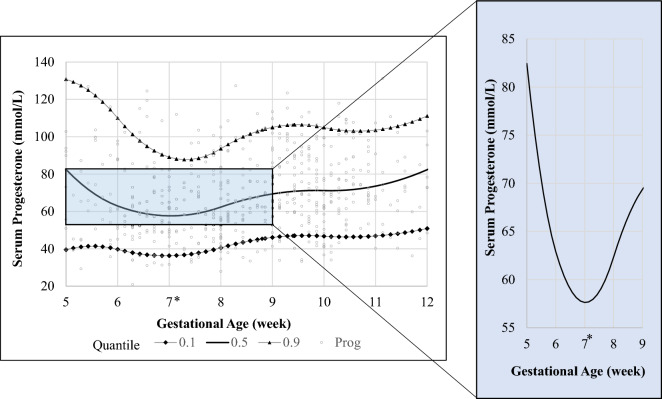
Gestational age-specific 10th, 50th and 90th percentile normative values of serum progesterone. Restricted cubic splines with knots at 6, 8 and 10 weeks were used to describe the shape of the serum progesterone trajectory across gestation. *Mean serum progesterone at gestational week 7 (63.4 nmol/L) compared against week 5 (75.0 nmol/L, p = 0.029), week 6 (66.9 nmol/L, p = 0.694), week 8 (67.7 nmol/L, p = 0.374) and week 9 (78.0 nmol/L, p < 0.001).

Gestational age-specific normative values and the trajectory of serum progesterone were established (Table 2; Fig. 2). Serum progesterone level started to decline after gestation week 5, reaching a nadir at week 7 [mean serum progesterone in week 5 = 75.0 nmol/L, compared to 66.9 nmol/L in week 6 (p = 0.057), and 63.4 nmol/L in week 7 (p = 0.029)]. It increases thereafter from gestation weeks 7–9 (mean serum progesterone in week 7 = 63.4 nmol/L, compared to 67.7 nmol/L in week 8 (p = 0.374) and 78.9 nmol/L in week 9 (p < 0.001).

**Table 2:** Gestational age-specific 2.5th, 10th, 50th, 90th and 97.5th percentile normative values of serum progesterone (nmol/L) in 590 women.

Gestational age (week)	n	2.5th percentile	10th percentile	50th percentile	90th percentile	97.5th percentile
5	44	35.8	42.3	69.0	116.0	139.5
6	87	31.3	36.2	59.4	108.6	124.4
7	76	32.9	41.4	61.5	89.9	103.5
8	103	34.6	40.5	62.6	99.6	127.2
9	126	40.3	49.0	73.6	106.3	158.6
10	91	38.1	48.2	72.4	101.8	117.9
11	33	36.2	45.4	70.8	107.4	183.4
12	30	48.5	63.5	91.0	116.7	169.4

### Determinants of serum progesterone

**Figure 3:**
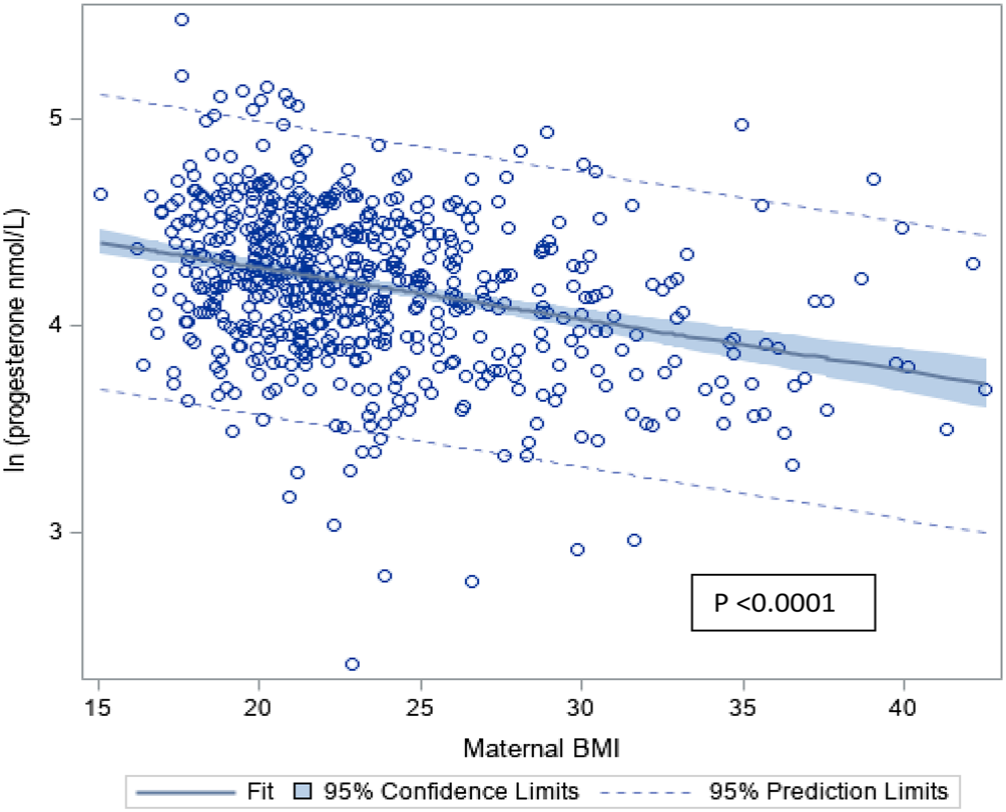
Relationship between maternal BMI and log serum progesterone levels, adjusted for maternal age, parity and gestational age.

Maternal characteristics that may potentially influence serum progesterone levels were investigated. Using a multiple linear regression analysis, maternal age, BMI, parity, gestational age and outcome of pregnancy at 16 weeks’ gestation were found to be the main determinants of serum progesterone levels at presentation, explaining 18.8% of the variability (Fig. 3; Supplementary Table S1).

Maternal age showed a small but significant positive correlation with serum progesterone levels (1.12, p < 0.0001). Nulliparous women had higher serum progesterone levels as compared to parous women (− 7.54 nmol/L, p = 0.0006). Women who were obese (BMI ≥ 27.5 kg/m^2^) had significantly lower serum progesterone levels as compared to those with normal BMI (mean serum progesterone difference of − 1.64 nmol/L, p < 0001).

### Serum progesterone levels in miscarriage

A subgroup of women who went on to have a miscarriage at 16 weeks or earlier were excluded from the main analyses. Serum progesterone levels in women who had miscarried by 16 weeks (48.2 ± 25.8 nmol/L) were significantly lower compared to women with viable pregnancies at 16 weeks (72.5 ± 26.3 nmol/L) for a mean difference (95% CI) of 24.2 (15.1, 33.5) (p < 0.001) (Supplementary Table S2).

## Discussion

We conducted a cross-sectional study to determine gestation age-specific normative values and determinants of serum progesterone through the first trimester of a viable low-risk pregnancy. To the best of our knowledge, this is one of the first studies to determine normative values of serum progesterone on a gestation week-by-week basis in the first trimester, which is the most critical stage of pregnancy. Having established a reference range, clinicians would be able to better identify women whose serum progesterone level deviate from the normal range, and correlate to clinical outcomes in future population-based studies. If abnormal gestational-age specific serum progesterone correlates with pathological outcomes, this will allow for more personalized medicine with closer monitoring and medical interventions for this group of women.

Even though serum progesterone is known to exhibit an overall increasing trend during pregnancy, we showed that it also follows an interesting trajectory of transient decline between weeks 6 and 8 of gestation with significantly lower levels seen in week 7. This decline corresponds to the period of luteal-placental shift. Progesterone is secreted by the corpus luteum, which only lasts for 14 days if a pregnancy does not occur. In early pregnancy, beta human chorionic gonadotropin (β-hCG) secreted by syncytiotrophoblasts maintains the corpus luteum, allowing it to produce progesterone until the placenta takes over its function at 7–9 weeks of gestation. Earlier studies defined this period of luteal-placental shift by demonstrating that lutectomy performed prior to week 7 resulted in an immediate progesterone drop with eventual abortion whereas lutectomy after week 9 resulted in pregnancy survival^9,10^. Furthermore, our data adds further evidence to studies by *Yoshimi* and *Jarvela*, in which a similar progesterone trajectory was described, albeit in a small cohort of 9 and 20 women, respectively^11,12^.

Luteal phase deficiency (LPD) is a condition of insufficient progesterone to maintain a normal secretory endometrium and allow for normal embryo implantation and growth^16^. This is one of many etiologies associated with early pregnancy loss. Two mechanisms have been proposed for LPD. The first and likely more common cause relates to the impaired function of the corpus luteum, resulting in insufficient progesterone and estradiol secretion. This impaired function can be the result of improper development of the dominant follicle destined to become the corpus luteum or aberrant stimulation of a normally developed follicle, leading to deficiencies in progesterone production. The second mechanism suggests an inability of the endometrium to mount a proper response to appropriate estradiol and progesterone exposure. In our study, it is worth highlighting that even though there was a transient decline in serum progesterone levels, it did not translate to adverse pregnancy outcomes such as miscarriages at 16 weeks of gestation. Our study highlighted the physiological decline in serum progesterone between weeks 6 and 8 in a normal low-risk group. Further work needs to be done to investigate pathological changes in serum progesterone during this crucial period in order to allow clinicians to better characterize LPD, which was previously impossible without a normal reference range.

We identified four important predictors of serum progesterone in the first trimester of a viable pregnancy—maternal age, BMI, gestational age and parity. Maternal BMI was found to be inversely correlated with serum progesterone, and that is consistent with many previous studies. This may be attributed to early biochemical alterations associated with obesity, leading to lower progesterone levels^17^. Reduced pulsatile luteinizing hormone amplitude and urine progesterone metabolites were seen in obese women as compared to those with normal body weight^18^. Adipocytokines have also been shown to negatively affect function of the corpus luteum^19^. Moreover, since progesterone is a lipid-soluble hormone, this equates to a higher volume of distribution, hence resulting in lower circulating serum progesterone levels.

Evidence regarding parity and its association with serum progesterone have been inconsistent, with most studies showing no significant differences in serum progesterone levels between the first and subsequent pregnancies^20^. We found that women who previously had at least one full-term pregnancy had lower serum progesterone levels as compared to primiparous women (mean serum progesterone difference of 5.4 ± 2.2 nmol/L, p = 0.011). This is in line with studies exploring long-term effects of a first pregnancy on the hormonal environment. In these studies, pregnancy-related hormones such as β-hCG^21–23^ and estradiol^24^, have been observed to decrease in pregnancies following the first child birth. Reduced β-hCG levels in turn leads to lower serum progesterone, as β-hCG is involved in sustaining the corpus luteum and progesterone production before the placenta takes over. The mechanism underlying this decline in certain pregnancy-related hormones is still largely unknown. It is postulated that prior full-term pregnancies may induce alterations in a woman’s hormonal milieu, such as altered maternal hormone metabolism, increased levels of binding proteins leading to reduced hormone bioavailability and modulation of hormone receptor expression^21^. Future studies can seek to delve further into this concept of hormonal alterations after a prior pregnancy, with possible implications on hormonal profiles in the critical first trimester period.

As part of our secondary aim, we compared the women with ongoing pregnancy to those who miscarried at or before 16 weeks. Serum progesterone levels in women who miscarried were significantly lower than those in women who had viable pregnancies, highlighting the important role of progesterone in maintaining early pregnancy as shown by prior literature and our previous study^2^.

The main strength of our study is that this is one of the first and largest population-based studies to determine gestation age-specific normative range of serum progesterone through the first trimester of viable low-risk pregnancy, which is the most crucial stage of a pregnancy. Furthermore, we were able to plot the trajectory of first trimester serum progesterone in this group. The main limitation is that the serum progesterone levels of women were measured at presentation across gestation, with inherent biological variability from woman to woman, instead of through a serial examination of serum progesterone in the same woman every week. However, adopting the latter study design would require study participants to return for weekly follow-ups and have blood tests performed at every visit, which would likely decrease compliance and increase dropout rates. In our study we did not demonstrate an association between race and progesterone levels. This may be limited by the study population, where about half (48.8%) are Chinese, while Malays, Indians and other races form a smaller minority (Table 1), which could reduce the generalizability of these study results, given that hormonal levels are shown to be affected by race^25^. This gives rise to the need for multi-ethnic studies to allow normative serum progesterone values to be individualized to specific patient populations.

## Conclusion

This is one of the largest studies describing the normative serum progesterone values across the first trimester of a low-risk viable pregnancy. We have demonstrated the physiological decline in serum progesterone corresponding to a luteal-placental shift between 6 and 8 weeks of gestation with a nadir at week 7. This study will form the basis for future work on pathological levels of serum progesterone that translates to adverse clinical outcomes, such as threatened or spontaneous miscarriage. Maternal age, BMI, parity, gestational age and outcome of pregnancy at 16 weeks’ gestation were also shown to be associated with serum progesterone levels. Larger studies investigating the trajectory and determinants of progesterone in the first trimester may shed light on the development of normative serum progesterone level, accounting for maternal age, BMI, parity and gestational age.

## Supplementary Information

Supplementary Information

## Data Availability

The datasets generated during and/or analysed during the current study are not publicly available due to confidentiality consent of the study but can be obtained from the corresponding author on reasonable request.
