# Supplementary material for: Gestational age-specific normative values and determinants of serum progesterone through the first trimester of pregnancy
Source: Sci Rep. 2021 Feb 18;11:4161. doi: 10.1038/s41598-021-83805-w (PMC7893162; doi:10.1038/s41598-021-83805-w)
Supplement: Supplementary file 1 — Supplementary Information [file 41598_2021_83805_MOESM1_ESM.docx]

**TITLE:** Gestational age-specific normative values and determinants of serum progesterone through the first trimester of pregnancy

**RUNNING TITLE:** Normative serum progesterone in first trimester

**AUTHORS:**

Chee Wai Ku^1,^ψ,^*^, Xiaoxuan Zhang^2,^ψ, Valencia Ru-Yan Zhang^3^, John Carson Allen^4^, Nguan Soon Tan^5,6^, Truls Østbye^7^, and Thiam Chye Tan^1,2^

^1^Department of Obstetrics and Gynecology, KK Women’s and Children’s Hospital, Singapore, 100 Bukit Timah Road, Singapore 229899, Singapore.

^2^Duke-National University of Singapore Medical School, Singapore, 8 College Road, Singapore 169857, Singapore.

^3^Yong Loo Lin School of Medicine, National University of Singapore, NUHS Tower Block Level 11, 1E Kent Ridge Road, 119228, Singapore.

^4^Centre for Quantitative Medicine, Duke-National University of Singapore Medical School, Singapore, 20 College Road, Academia, Singapore 169856, Singapore.

^5^Lee Kong Chian School of Medicine, Nanyang Technological University Singapore, 11 Mandalay Road, 308232, Singapore.

^6^School of Biological Sciences, Nanyang Technological University Singapore, 60 Nanyang Drive, 637551, Singapore.

^7^Department of Community and Family Medicine, Duke University Medical Center, 2200 West Main Street, Durham, North Carolina 27710, USA.

**Supplementary Table S1.** Multiple regression analysis on serum progesterone levels at 5-12 weeks gestational age by pregnancy outcome at 16 weeks (r^2^ = 0.22)

| **Parameter** | **Coeff.** |  | **95% CL** | |  | **P-values** |
| --- | --- | --- | --- | --- | --- | --- |
|  |  |  | **LCL** | **UCL** |  | **Prog** |
| Intercept | 51.68 |  | 31.35 | 72.01 |  | <0.0001 |
| Age | 1.12 |  | 0.58 | 1.67 |  | <0.0001 |
| Ethnicity (ref=Others) |  |  |  |  |  |  |
| Chinese | 0.89 |  | -4.66 | 6.45 |  | 0.7529 |
| Malay | 1.10 |  | -6.23 | 8.43 |  | 0.7687 |
| Indian | 1.06 |  | -6.94 | 9.05 |  | 0.7948 |
| BMI | -1.64 |  | -2.10 | -1.18 |  | <0.0001 |
| Parity ≥1 | -7.54 |  | -11.82 | -3.27 |  | 0.0006 |
| Gestation age | 3.19 |  | 2.08 | 4.30 |  | <0.0001 |
| 16-week miscarriage | -18.82 |  | -27.14 | -10.50 |  | <0.0001 |
| History of miscarriages | -2.74 |  | -8.00 | 2.51 |  | 0.3050 |
| TOP | 2.19 |  | -4.89 | 9.26 |  | 0.5443 |
| Smoker | -6.86 |  | -17.22 | 3.50 |  | 0.1937 |
| Alcohol | -30.42 |  | -75.24 | 14.40 |  | 0.1830 |

**Supplementary Table S2.** Serum progesterone levels at 5-12 weeks gestational age by pregnancy outcome at 16 weeks

|  | **n** | **Mean serum progesterone (nmol/L)** | **p-value**  **(t-test)** |
| --- | --- | --- | --- |
| **Outcome at 16 weeks** | | | |
| Pregnancy | 590 | 72.5 (26.3) | <0.001 |
| Miscarriage | 33 | 48.2 (25.8) |  |
